# Genome Sequences of Two Mycobacterium tuberculosis Isolates from Asian Elephants in Nepal

**DOI:** 10.1128/MRA.00614-21

**Published:** 2021-09-09

**Authors:** Sarad Paudel, Evan P. Brenner, Syeda A. Hadi, Yasuhiko Suzuki, Chie Nakajima, Toshio Tsubota, Kamal Prasad Gairhe, Bhagwan Maharjan, Srinand Sreevatsan

**Affiliations:** a Pathobiology & Diagnostic Investigation Department, College of Veterinary Medicine, Michigan State University, East Lansing, Michigan, USA; b Division of Bioresources, Hokkaido University International Institute for Zoonosis Control, Sapporo, Japan; c International Collaboration Unit, Hokkaido University International Institute for Zoonosis Control, Sapporo, Japan; d Laboratory of Wildlife Biology and Medicine, Graduate School of Veterinary Medicine, Hokkaido University, Sapporo, Japan; e Department of National Parks and Wildlife Conservation, Chitwan National Park, Chitwan, Nepal; f National Tuberculosis Control Center (NTCC), Thimi, Bhaktapur, Nepal; Loyola University Chicago

## Abstract

This report describes the genome sequences of two Mycobacterium tuberculosis isolates, S1 and S3, recovered from Asian elephants in Nepal. These genome sequences will enhance our understanding of the genomic epidemiology of Mycobacterium tuberculosis in Asian elephants.

## ANNOUNCEMENT

Asian elephants (Elephas maximus) are an endangered species (1) with fewer than 50,000 individuals remaining worldwide (2). Tuberculosis (TB) in wild and captive elephants further jeopardizes the survival of the species (3).

Lung lesions reminiscent of granulomas and suspected to be tuberculosis were collected at necropsy of two captive Asian elephants from Chitwan National Park, Nepal. Lung tissue samples were processed for inoculation into Löwenstein-Jensen (L-J) medium, incubation at 37°C for ∼8 weeks, and DNA extraction using the GenoType DNA isolation kit (Hain Lifescience GMBH, Nehren, Germany) from a slant culture at the TB laboratory of the German Nepal Tuberculosis Project (GENETUP) in Kathmandu, Nepal (4). Confirmation and genotyping for Mycobacterium tuberculosis, including multiplex PCR, *gyrB* sequencing, spoligotyping, and variable-number tandem repeat (VNTR) analysis, were performed at the Hokkaido University Research Center for Zoonosis Control, Hokkaido, Japan, as described (4). Genomic DNA was submitted to Novogene for whole-genome sequencing.

The NEBNext DNA library prep kit (New England BioLabs, Ipswich, MA) was used to generate paired-end (2 × 150-bp) libraries, which were quality checked using Qubit 2.0, the Agilent 2100 Bioanalyzer, and quantitative PCR (qPCR) prior to whole-genome sequencing (WGS) on the Illumina NovaSeq 6000 platform. Novogene’s custom software trimmed adapters and discarded paired-end reads containing contaminant adapter sequences, pairs of reads containing more than 10% uncertain nucleotide calls, and pairs containing Phred scores less than or equal to 5. The reads were analyzed using Kraken2 v2.1.2 to eliminate contaminating sequences (5), and 92.7% of S1 and 99.12% of S3 reads were mapped to the Mycobacterium tuberculosis complex (MTBC) using the Kraken2 default parameters and database. MTBC-classified reads were separated using the extract_kraken_reads.py script from KrakenTools (Jennifer Lu, KrakenTools). HISAT2 v2.2.1 was used to determine the coverage of reads relative to M. tuberculosis H37Rv (GenBank accession number NC_000962.3) (6). Genome assembly was performed *de novo* using Shovill v1.1.0 (-minlen 300 –gsize 4.41M) to reject contigs shorter than 300 bp (7). RagTag v1.1.1 corrected and scaffolded the output using default parameters and M. tuberculosis H37Rv (NC_000962.3) as reference (8). The scaffolds were evaluated using QUAST v5.02 (9) and annotated using the NCBI Prokaryotic Genome Annotation Pipeline (PGAP) v5.1 (10). NCBI flagged potential adapter contamination in S3 only, so Kraken2-cleaned S3 reads were processed using Trimmomatic v0.39 (trimming TruSeq3 adapters, parameters: 2:30:10: LEADING:3 TRAILING:3 MINLEN:36) (11) before assembly and successful PGAP resubmission.

Genome sequencing of strain S1 yielded a total of 6,804,775 unfiltered reads. Shovill assembly produced 26 contigs with an *N*_50_ value of 4,383,806 bp. The total sequence length of the strain S1 genome is 4,475,258 bp, with a GC content of 65.57% and ∼359× coverage. The NCBI PGAP identified 4,164 coding DNA sequences (CDSs), 45 tRNAs, 3 noncoding RNAs (ncRNAs), and 198 pseudogenes in strain S1.

Similarly, sequencing of strain S3 resulted in 6,838,686 total unfiltered reads. Shovill assembly produced 19 contigs with an *N*_50_ value of 4,382,763 bp. The total sequence length of the strain S3 genome is 4,398,381 bp, with a GC content of 65.59% and ∼385× coverage. The NCBI PGAP identified 4,069 CDSs, 45 tRNAs, 3 ncRNAs, and 147 pseudogenes. This study reports the M. tuberculosis genome sequences from endangered Asian elephants in Nepal and highlights the potential for whole-genome sequencing in genomic epidemiological studies of TB in elephants.

### Data availability.

The raw reads are available under NCBI BioProject accession number PRJNA729522. The raw reads are available under SRA accession numbers SRX10859885 (strain S1) and SRX10859886 (strain S3). The post-PGAP sequences are available under GenBank accession numbers JAHCQW000000000 (strain S1) and JAHCQV000000000 (strain S3).
